# ELONGATED HYPOCOTYL5 Regulates Resistance to Root-Knot Nematode by Modulating Antioxidant System and Jasmonic Acid in *Cucumis sativus*

**DOI:** 10.3390/antiox14060679

**Published:** 2025-06-03

**Authors:** Fusheng Ma, Juanqi Li, Mengwei Huang, Mengyan E, Dandan Cui, Guoxiu Wu, Shengli Li, Yang Li

**Affiliations:** College of Horticulture, Henan Agricultural University, Zhengzhou 450002, China; mafusheng2810@163.com (F.M.); ljq881229@henau.edu.cn (J.L.); hmw0843@163.com (M.H.); m17630691690@163.com (M.E.); cuidandan@henau.edu.cn (D.C.); guoxiuwu@henau.edu.cn (G.W.)

**Keywords:** *Cucumis sativus*, root knot nematodes, CsHY5, hormones, antioxidant system

## Abstract

Root-knot nematodes (RKNs), specifically *Meloidogyne incognita*, are notoriously difficult to eliminate as endophytic nematodes, and cause severe damage to various plants. Cucumber (*Cucumis sativus*), which is a cash crop widely grown across the world, is often infected by RKNs. ELONGATED HYPOCOTYL5 (HY5), a member of the bZIP transcription factor family, plays a vital role in hormone, nutrient, abiotic stress, biotic stress, and oxygen species (ROS) signaling pathways. However, the involvement of HY5 in the defense against RKNs has rarely been reported. The present study initially explored the response of CsHY5 to RKNs. The results indicated that the *hy5* mutant had a higher number of nematodes and galls in the root system and exhibited a higher susceptibility to RKNs compared with the wild type (WT). Particularly, the root-knot nematodes in *hy5* plants completed their life cycle more quickly and produced more eggs. The activities of defense-related hormones and antioxidant enzymes were measured, and the results indicated that JA, jasmonoyl-isoleucine (JA-Ile), abscisic acid (ABA), peroxidase (POD), and ascorbate peroxidase (APX) were significantly elevated in the wild type, but were not induced or decreased in the mutant. Through transcriptome sequencing analysis and quantitative real-time PCR (qRT-PCR), it was found that when RKNs infect plants, the key genes of jasmonic acid (JA) synthesis, *CsAOC* and *CsAOS*, as well as the key gene of the antioxidant system, *CsPOD*, were all significantly induced. Nevertheless, this induction effect disappeared in the *hy5* mutant. Generally, CsHY5 plays a role in the response of cucumber to RKNs, and its deletion increases the sensitivity of cucumber to RKNs. These results suggest that *CsHY5* may affect the resistance of cucumber to RKNs by affecting antioxidant enzyme activities and hormone content.

## 1. Introduction

It is estimated that the annual global economic losses in crop yield caused by plant-parasitic nematodes reach USD 173 billion, despite the phytosanitary measures taken to control nematodes [1]. Root-knot nematodes (RKNs; *Meloidogyne* spp.) are extensively researched as they are endophytic resident nematodes, presenting significant challenges in terms of control [2]. RKN species mainly comprise *Meloidogyne incognita*, *Meloidogyne javanica*, and *Meloidogyne arenaria*. Among them, *Meloidogyne incognita* is a widespread species and is regarded as one of the most damaging crop parasites worldwide [3]. RKNs infect a variety of commercially significant crops, such as cucumber, rice, melon, and tomato [4,5,6]. RKNs infect host plants in soil as second-stage juveniles (J2s) [7]. J2s can penetrate the root cap and enter the root interior but are unable to penetrate the Casparian strip in the root. Thus, J2s eventually move around the vascular bundle and establish feeding sites [7,8], relying on the host root system to supply nutrients. J2s undergo three molts to develop into vermiform male and pear-shaped female adults [2,7,9]. The development of J2s leads to abnormal cell division near the feeding site, cell enlargement, and eventually the formation of multiple giant cells [10,11,12]. Since the giant cells are encompassed within the intricate xylem network, the phloem of the plant encloses the giant cells and multiplies rapidly, eventually forming a gall referred to as a root-knot on the root surface, which is the typical symptom of RKN disease [13,14,15]. Until now, the commonly used methods for controlling RKNs include chemical control, physical control, biological control, and screening of resistant varieties, among which chemical control has the advantages of quick effect and simple application methods. However, chemical control causes serious environmental pollution, so it is particularly important to explore a safe and effective control technology.

In contrast to animals, plants do not have mobile defense cells and somatic adaptive immune systems. Instead, they rely on innate immunity and systemic signals from infection sites to adapt to adverse survival conditions (such as drought, salinity, extreme temperatures, heavy metal contamination, pathogen infection, and feeding by herbivorous animals, etc.) [16,17,18,19]. There are essentially two types of immune system in plants, one is PAMP-triggered immunity (PTI) and the other is effector-triggered immunity (ETI) [16]. Plants rely on PTI- and ETI-induced downstream immune responses (plant hormones, ROS, defense-related genes) to resist root-knot nematodes [20]. PTI is of crucial significance for the defense of plants against RKNs. During PTI, a variety of induced defense responses take place, including morphological, physiological and molecular alterations. For instance, upon RKNs invasion, reactive oxygen species (ROS) are quickly accumulated in the invaded cells and cell walls [21]. In *Arabidopsis thaliana*, the NADPH oxidases, namely, RbohD and RbohF, produce ROS when infected by RKNs, thereby restricting the death of infected plant cells and promoting the formation of nurse cells [22]. On the other hand, during the infection of plants by RKNs, a variety of phytohormones are implicated in the interactions between plants and RKNs, such as JA, SA, ethylene, ABA, brassinosteroids (BR), and strigolactones (SLs) [23,24,25,26,27,28]. In particular, RKN resistance is largely dependent on JA synthesis in shoots [29]. Plants often accumulate jasmonates (JAs) in response to herbivores, thereby inducing defense responses [30]. In tomato, exogenous spraying of JA significantly enhanced the expression level of the nematode-resistance marker gene *Mi*, reducing root-knot nematode diseases in plants [31]. Recent studies revealed that JA negatively regulates RKN susceptibility via the root exudates of tomato plants [32]. In addition, the JA defense-dominated genotype exhibits stronger resistance to RKNs compared with that of the wild-type *Castlemart* and the JA-deficient mutant *spr2* [33].

Light is one of the indispensable conditions for the growth and development of plants. Meanwhile, light plays a significant role in plants’ resistance to pathogen infection, and this role is mainly achieved by regulating hormone synthesis and the antioxidant system [34,35,36]. Supplying red light to tomatoes at night induces systemic resistance against RKNs, which is partly dependent on the JA and SA defense pathways [37]. Islam et al. found that red light could effectively induce systemic disease resistance against RKNs and *Pseudomonas syringae* pv. Tomato *DC 3000* [38]. In the presence of red light, CmWRKY42 activated by red light directly binds to the promoter of *CmICS*, activates its expression, and promotes the accumulation of SA, thereby enhancing the resistance of oriental melon to powdery mildew [39]. In watermelon, the accumulation of H_2_O_2_ increased in red light-treated RKN-plants, indicating that the H_2_O_2_ signal plays a key role in mediating the systemic defense induced by red light [34]. In addition to red light, blue light alleviates the degradation of HRT, enabling plants to be resistant to *Turnip crinkle virus* [40]. The expression of the SA-induced pathogenesis-related gene *PR-1* is decreased in *cry*1 mutants but increased in *cry1-ox* plants. Thus, the blue light photoreceptor *CRY1* positively regulates inducible resistance to *P. syringae* [41].

At present, a large amount of research has been conducted on how plants enhance their resistance to adverse external environments after being exposed to different light conditions. Among them, ELONGATED HYPOCOTYL5 (HY5), as a key central factor connecting light and plant resistance, has played an important role [42]. HY5 belongs to the bZIP family of transcription factors and can regulate the expression of about one-third of the genes in the genome, which are directly or indirectly involved in multiple processes such as plant growth, hormone response, and environmental response [43,44]. HY5 can improve the tolerance of Arabidopsis to cold stress by binding to the cold tolerance gene *CAB1* [45]; *SlHY5* affected the cold tolerance of tomato by regulating the content ratio of ABA and GA [46]. HY5/HYH directly bind to the promoters of the reactive oxygen species (ROS) signal-related genes such as APX2, ZAT10, SIB1, ERF4, and NDB2. Furthermore, red light leads to the expression of HY5, which directly activates the Enhanced Disease Susceptibility 1 (*EDS1*) gene and strengthens the defense against pathogens [47]. A recent study revealed a critical mechanism through which red light induces SA accumulation by regulating CaHY5-mediated *CaPAL3* and *CaPAL7* expression, leading to enhanced resistance to *P. capsici* infection [48]. However, reports on whether HY5 can regulate the resistance of plants to RKNs are scarce, especially in cucumber (*Cucumis sativus*), which are highly susceptible to RKNs and widely cultivated.

Cucumber is frequently infected by RKNs, resulting in significant economic losses during the cultivation process [49]. Yield losses due to RKNs could reach up to 88% under facility cultivation conditions [50]. In the production process, chemical methods are still commonly used to control RKN density. However, they have certain adverse effects on the soil environment. If plant resistance can be utilized to control pests and diseases, it would be the most economical, environmentally friendly, and effective approach [51]. As an important light response factor, HY5 can integrate light signals and defense pathways in plants, but the role of CsHY5 (XM_004138683) in cucumber RKNs has been rarely reported. Revealing the relationship between the *HY5* gene and the plant’s resistance to RKNs in advance is helpful to provide a theoretical basis for the future regulation of plant nematode resistance by light quality. In this study, we investigated the role of *CsHY5* in cucumber root infection by RKNs. The *hy5* mutant was found to be more susceptible, with a great number of nematodes entering the *hy5* mutant root system and faster development to the female stage. By measuring antioxidant enzyme activities and several hormones, we found that *hy5* mutants were unable to activate antioxidant enzyme activities when RKNs invaded their roots, which was similar to the hormones. We also found that the magnitude of changes in the accumulation of JA content was the highest during RKN invasion of roots, indicating that JA-induced defense pathways might be the most crucial in cucumber defense against RKNs.

## 2. Materials and Methods

### 2.1. Cucumber Transformation

**Figure 1:**
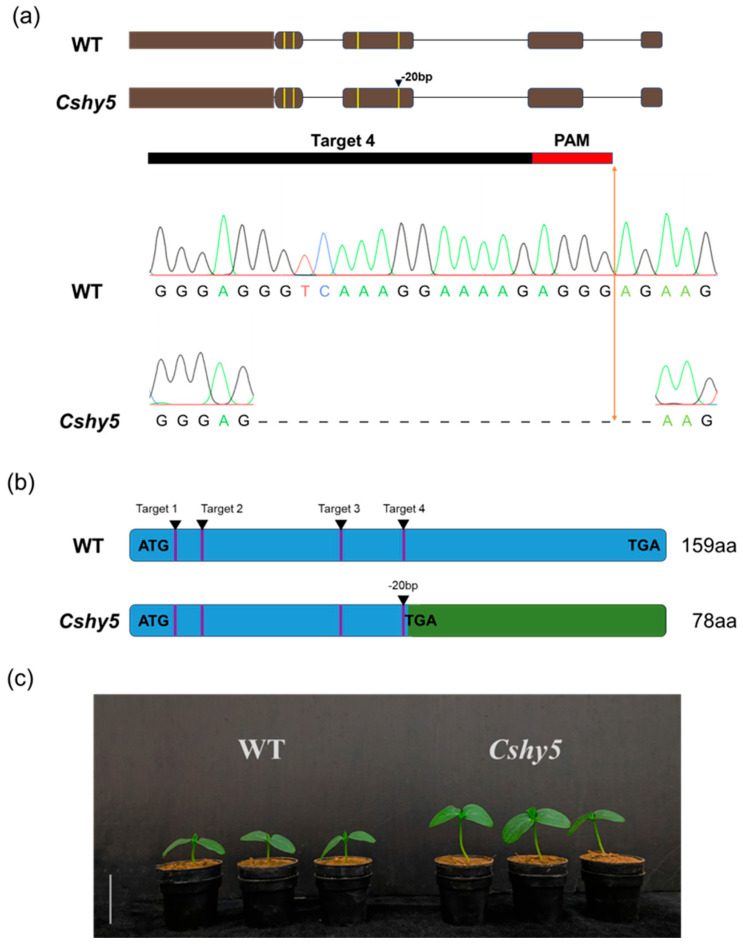
Knockout of *CsHY5* by CRISPR/Cas9. (**a**) The sanger sequencing chromatogram data of the transgenic homozygous mutant *Cshy5*, where the yellow lines represent the target sites, namely, the first, second, third, and fourth from left to right, with a 20 bp base deletion at the fourth target. The long line represents the target sequence, and the short line represents the PAM region. (**b**) The *Cshy5* homozygous mutant lines have a 20 bp base deletion at the fourth target site, resulting in the premature appearance of the stop codon TGA and premature translation termination, which leads to protein truncation. (**c**) Phenotypic map of wild type and *Cshy5* plants. The scale bar is 4 cm.

To generate the construct used for CRISPR/Cas9-edited plants of CsHY5 (XM_004138683), the specific sgRNA target sites were selected by the sgRNA design web (http://www.rgenome.net/cas-designer/, accessed on 17 December 2019). The PCR fragment harboring four target sites was amplified using eight partially overlapping primers and then inserted in the *Bsa I* site of the binary CRISPR/Cas9 vector pKSE402. The recombinant plasmids were transformed into the XTMC using a cotyledon transformation method [52]. The sequencing results showed that there was a stable base loss in the target sequence. As shown in Figure 1, there was a loss of twenty bases at the end of the fourth single-guide RNA target site. The phenotypic analysis showed that the hypocotyls of *Cshy5* mutants were significantly longer than those of the WT, indicating that the function of CsHY5 in regulating hypocotyl development was disrupted and the plants were successfully edited (Figure 1c). The primers are listed in Supplementary Table S1.

### 2.2. Plant Material and Growth Condition

In this study, cucumber *Cshy5* mutants and the corresponding WT (XTMC) were employed. Seeds were soaked in 55 °C water for 5 h, then placed in Petri dishes with wet filter paper, and subsequently placed in a dark incubator at 28 °C for 24 h to expedite germination. After germination, each germinated seed was sown in a nutrient bowl (5 cm × 5 cm × 4 cm) containing sterilized garden soil. These seeds were grown in an artificial climate chamber with controlled environmental conditions (28 °C:24 °C, day–night, temperature; 60% relative humidity; 12 h:12 h, light–dark cycle).

### 2.3. Propagation and Inoculation of Meloidogyne Incognita

*Meloidogyne incognita* was provided by the China Agricultural University and propagated with cabbage in the artificial climate chamber of the College of Horticulture, Henan Agricultural University. Approximately 40 days after the cabbage was inoculated with nematodes, the roots were collected, the eggs were picked and placed in a Petri dish with distilled water. To prevent contamination with impurities, several rinses with sterilized distilled water were conducted. Second-stage juveniles (J2s) of *Meloidogyne incognita* in water were gathered 7 days after incubation. When cucumber seedlings grew to the single leaf stage, 100 J2s were inoculated per plant, while control plants were simulated inoculation using sterilized water.

### 2.4. Susceptibility Assessment

To assess the susceptibility of the plants to nematodes, nematode-infected roots were collected 3, 7, and 14 days after inoculation (DAI), washed with water, and observed by acid fuchsin staining [53]. After an overnight period, the number of root knots as well as the number of nematodes at different instars were counted under a stereomicroscopy. Six biological replicates were set for each treatment per sampling in the experiment.

### 2.5. RNA-Seq Experimental Method

Uninfected and infected leaves from WT and *Cshy5* plants were collected at 7 DAI and immediately snap-frozen in liquid nitrogen for transcriptome sequencing. Total RNA was isolated using Trizol Reagent (Invitrogen Life Technologies, Waltham, MA, USA), followed by qualification and quantification: (1) RNA purity and concentration were assessed using a NanoDrop spectrophotometer (Thermo Scientific, Waltham, MA, USA) and Qubit 4.0; (2) RNA integrity and quantity were evaluated via the Agilent 2100/4200 system (Santa Clara, CA, USA).

For library preparation, three micrograms of qualified RNA served as input material: mRNA was first purified from total RNA using poly-T oligo-attached magnetic beads, then fragmented with divalent cations under elevated temperature in fragmentation buffer. First-strand cDNA was synthesized using random hexamers, followed by second-strand synthesis with buffer, dNTPs, RNase H, and DNA polymerase I. Double-stranded cDNA underwent end repair and 3′-end adenylation, after which HieffNGS^®^ DNA Selection Beads (Yeasen, Shanghai, China) were used for purification and fragment size selection. The purified products were amplified and enriched by PCR, and Qubit was employed for quantitation.

Following library preparation, the sequencing libraries were sequenced on the DNBSEQ-T7 (PE150 model) or Illumina Novaseq platform at Bioyi Biotechnology Co., Ltd. (Wuhan, China).

### 2.6. RNA Isolation and Complementary DNA Synthesis

The whole leaves and roots of infected and non-infected plants were collected. Each treatment comprised three biological replicates. Subsequently, these samples were rapidly frozen in liquid nitrogen and then ground into a fine powder using a sterilized mortar and pestle, and the plant powder material was dispensed into 2 mL enzyme-free centrifuge tubes as per the instructions. Total RNA was extracted from 0.1 g samples using the Quick RNA Isolation Kit (Huayueyang, Beijing, China) in accordance with the manufacturer’s protocol. Total RNA (1 μg) was reverse transcribed to synthesize cDNA using HiScript Ⅱ Q RT superMix for qPCR (+gDNA wiper) (Vazyme, Nanjing, China) following the manufacturer’s protocol. The cDNA was diluted two-fold using RNase-free ddH_2_O.

### 2.7. Quantitative Real-Time PCR

The quantitative PCR reactions were performed in a 20 μL system, which contained 10 μL ChamQ Universal SYBR qPCR Master Mix (Vazyme, Nanjing, China), 0.4 μL (10 μM) forward primer, 0.4 μL (10 μM) reverse primer, 2 μL cDNA, and 7.2 μL ddH_2_O. The quantitative real-time (qRT) PCR was conducted using a CFX Connect Real-Time PCR Detection System (Bio-Rad, Hercules, CA, USA). The PCR conditions included predenaturation at 95 °C for 3 min, followed by 40 cycles of denaturation at 95 °C for 30 s, annealing at 60 °C for 30 s, and extension at 72 °C for 30 s. A melt-curve analysis was performed for each sample run using the Bio-Rad default parameters (95 °C for 10 s, 65 °C–95 °C in 0.5 °C increments) resulting in one peak. Each biological sample contained three technical replicates and was normalized to the housekeeping gene *Csactin*. Relative expression was based on the 2^−ΔΔCT^ method [54]. The gene-specific primers are detailed in Supplementary Table S1.

### 2.8. Determination of Antioxidant Enzyme Activity

On the 21st day of root-knot nematode infection in cucumber, 0.2 g of leaf was pulverized in 1 mL of chilled enzyme buffer, composed of 25 mM HEPES, 0.2 mM ethylenediamine tetraacetic acid (EDTA), 2 mM ascorbic acid (AsA), and 2% polyvinylpolypyrrolidone (*w*:*v*) at pH 7.8. These were then homogenized with a swirl, centrifuged at 12,000× *g* for 20 min, and the supernatants obtained were utilized for assessing POD, superoxide dismutase (SOD), catalase (CAT), and APX enzymes. The methodologies and conditions for these measurements were based on a previously established method that was described by Liu et al. [55]. The activity of each enzyme was expressed based on protein content. The protein content was determined according to the method of Bradford [56], as follows: 0.2 g of leaf tissue was ground into powder with 1.5 mL of pre-cooled distilled water, followed by homogenization via rotational mixing. The homogenate was centrifuged at 12,000× *g* for 10 min to obtain the supernatant. Subsequently, 100 μL of the supernatant was mixed with 900 μL of distilled water and 5 mL of 0.117 mM Coomassie Brilliant Blue G-250 solution. After allowing the mixture to stand for 2 min, the absorbance was measured at 595 nm. Bovine serum albumin (BSA) was used as the standard to quantify the protein concentration.

To analyze the activity of POD (EC 1.11.1.7), at 25 °C, we combined 100 μL of the enzyme solution with 1700 μL of 25 mM PBS (pH 7.0, including 0.1 mM EDTA), 100 μL of 10 mM H_2_O_2_, and 100 μL of 1% guaiacol. Then, POD activity was estimated at 470 nm by its ability to convert guaiacol to tetraguaiacol (ε = 26.6 mM^−1^ cm^−1^). Regarding the analysis of APX (EC 1.11.1.11) activity, at 25 °C, we mixed 100 μL of the enzyme solution with 1700 μL of 25 mM PBS (pH 7.0, containing 0.1 mM EDTA), 100 μL of 20 mM H_2_O_2_, and 100 μL of 5 mM AsA. H_2_O_2_-dependent oxidation of ascorbate was followed by a decrease in the absorbance at 290 nm (ε = 2.8 mM^−1^ cm^−1^). For the determination of CAT (EC 1.11.1.6) activity, the reaction mixture in a total volume of 2 mL contained 25 mM sodium phosphate buffer (pH 7.0), 10 mM H_2_O_2_. The reaction was initiated by the addition of 100 μL of enzyme extract and activity was determined by measuring the initial rate of disappearance of H_2_O_2_ at 240 nm (ε = 39.4 mM^−1^ cm^−1^) for 60 s. For SOD (EC 1.15.1.1) activity analysis, we combined 50 μL of the enzyme solution with 3 mL of the reaction solution. The reaction solution consisted of 50 mM PBS (pH 7.8), 15 mM methionine, 65 mM nitroblue tetrazolium (NBT), 2 μM riboflavin, and 0.1 mM EDTA. After exposing the mixture to light at 25 °C under 4000 lx for 15 min, we measured the absorbance at 560 nm. One unit of SOD activity was defined as the quantity of enzyme needed to inhibit 50% of the NBT reduction rate at 560 nm.

### 2.9. Determination of Plant Hormones

Leaves of the WT and *Cshy5* plants, both infected and uninfected with RKNs, were collected at 7 DAI and snap-frozen in liquid nitrogen. HPLC-grade acetonitrile (ACN) and methanol (MeOH) from Merck (Darmstadt, Germany), MilliQ water (Millipore, Bradford, PA, USA), standards from Olchemim Ltd. (Olomouc, Czech Republic) and isoReag (Shanghai, China), and acetic/formic acids from Sigma-Aldrich (St Louis, MO, USA) were used. Standard stock solutions (1 mg/mL in MeOH) were stored at −20 °C and diluted with MeOH to working concentrations prior to analysis. Fresh plant samples were harvested, snap-frozen in liquid nitrogen, and stored at −80 °C; 50 mg of sample was weighed into a 2 mL microtube, frozen in liquid nitrogen, and dissolved in 1 mL methanol/water/formic acid (15:4:1, *v*/*v*/*v*) with 10 μL internal standard mixed solution (100 ng/mL). The mixture was vortexed for 10 min, centrifuged at 15,294× *g* for 5 min at 4 °C, and the supernatant was transferred, evaporated to dryness, redissolved in 100 μL 80% methanol (*v*/*v*), and filtered through a 0.22 μm membrane for LC-MS/MS analysis.

Sample extracts were analyzed using a UPLC-ESI-MS/MS system (UPLC: ExionLC™ AD; MS: QTRAP^®^ 6500+, Sciex, Framingham, MA, USA) with a Waters ACQUITY UPLC HSS T3 C18 column (100 mm × 2.1 mm i.d., 1.8 µm). The solvent system comprised water with 0.04% acetic acid (A) and acetonitrile with 0.04% acetic acid (B), following a gradient program: 5% B (0–1 min), increasing to 95% B (1–8 min), holding at 95% B (8–9 min), and reverting to 5% B (9.1–12 min) at a flow rate of 0.35 mL/min, 40 °C column temperature, and 2 μL injection volume.

The QTRAP^®^ 6500+ LC-MS/MS System (Sciex), equipped with an ESI Turbo Ion-Spray interface under Analyst 1.6.3 software control, operated in positive/negative ion modes with set parameters: source temperature 550 °C, ion spray voltage 5500 V (positive)/−4500 V (negative), and curtain gas (CUR) 35 psi. Phytohormones were analyzed via scheduled multiple reaction monitoring (MRM), with Multiquant 3.0.3 software for metabolite quantification. Mass spectrometer parameters (declustering potentials, collision energies) were optimized for individual MRM transitions, and specific MRM transitions were monitored per period based on metabolite elution profiles.

### 2.10. Statistical Analysis

All data were recorded using Microsoft Excel 2016. Statistical analysis was performed using SPSS statistics, version 25.

## 3. Results

### 3.1. Loss of CsHY5 Reduced Plant Resistance to Root-Knot Nematodes

**Figure 2:**
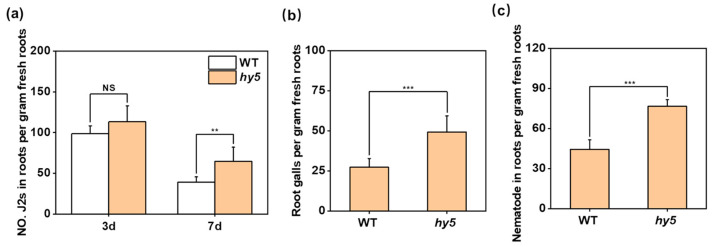
The loss of CsHY5 affects the susceptibility of plants to RKNs. (**a**) Number of J2s within the roots of WT and *Cshy5* mutants at 3 DAI and 7 DAI. (**b**) Number of root galls in WT and *Cshy5* mutants at 7 DAI. (**c**) Number of total nematodes in the roots of wild and *Cshy5* mutants at 7 DAI. The results are presented as the means ± SD; n = 6. Asterisks represent significant differences between *Cshy5* mutants and WT (NS, no significance; **, *p* < 0.01; ***, *p* < 0.001; Student’s *t*-test).

To explore the potential mechanism by which *CsHY5* regulates the sensitivity of cucumber root-knot nematodes, we employed CRISPR/Cas9 technology to establish *Cshy5* mutants on the XTMC background. Subsequently, we conducted nematode infection experiments using the *Cshy5* mutant and WT (XTMC). After inoculation with RKNs, the amount of J2s invading the *Cshy5* mutant roots at 3 DAI was higher than that of the WT, but not significantly so (Figure 2a). After a certain period of development, young root knots could be observed on the root system at 7 DAI. Through statistical analysis, we found that the number of galls on the roots of *Cshy5* mutant plants was significantly higher than that of WT plants (Figure 2b), and simultaneously, the number of J2s in the roots of *Cshy5* plants was significantly higher than that of WT plants (Figure 2a). The total number of nematodes in the roots of the *hy5* mutant was also higher than that of the WT (Figure 2c).

**Figure 3:**
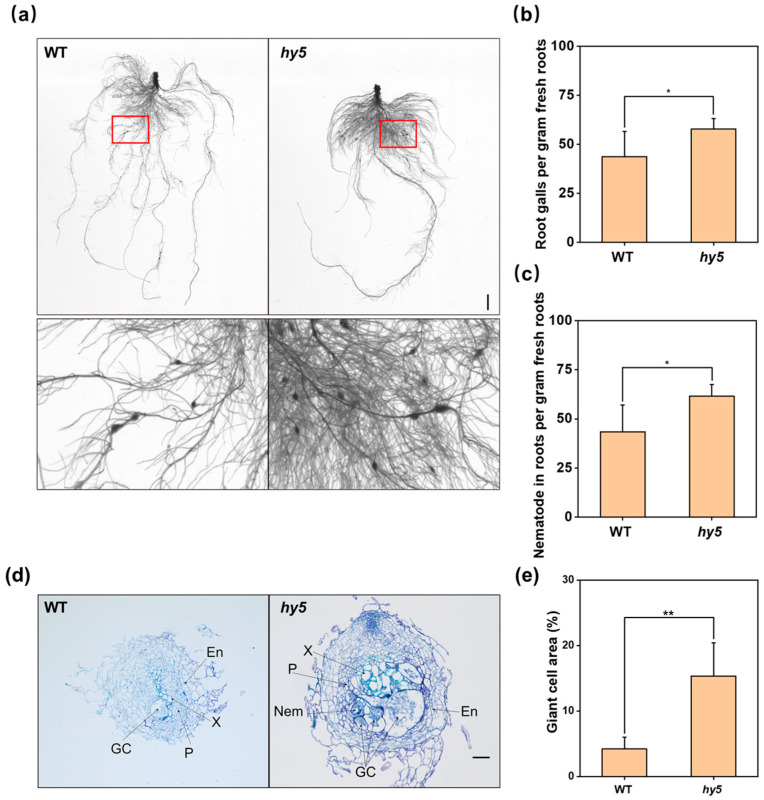
Sensitivity of *Cshy5* mutants to nematodes compared with WT at 14 DAI. (**a**) Scans of *Cshy5* mutants and WT roots at 14 DAI. (**b**) Number of root galls in *Cshy5* mutants versus WT plants at 14 DAI. (**c**) Number of total nematodes in the roots of *Cshy5* mutants and WT at 14 DAI. (**d**) Anatomical structure of root knots in WT and *Cshy5* mutants root systems. (**e**) Area occupied by giant cells in WT and *Cshy5* mutants root knots. In (**b**,**c**), the results are presented as the means ± SD; n = 6. In (**e**), the results are presented as mean ± standard deviation; n = 18. The scale bar is 2 cm in length in (**a**). The scale bar is 100 μm in length in (**d**). Asterisks represent significant differences between *Cshy5* mutants and WT (Nem, nematode; GC, giant cell; En, endodermis; P, phloem; X, xylem) (*, *p* < 0.05; **, *p* < 0.01; Student’s *t*-test).

To further investigate the role of *CsHY5* during RKN infection of cucumber, we carried out a sensitivity evaluation at 14 DAI after infection, and the results showed that the number of galls in the roots of *Cshy5* mutant plants was significantly higher than that of WT plants at 14 DAI. The number of total nematodes in the roots exhibited the same trend (Figure 3a–c). Through the sensitivity evaluation, we found that the deficiency of *CsHY5* decreased the resistance of cucumber to RKNs, which was confirmed by the important role of *CsHY5* in the response of cucumber to RKN infection, whether it was the J2 invasion at the early stage of infection (7 DAI), or the number of galls and nematodes in the root at 7 and 14 DAI. When comparing the root knot morphology in WT and *Cshy5* roots, we observed that most of the root knots appeared larger in *Cshy5* roots. To better explain this phenomenon, we examined the anatomical structure of the root knots of the two, and we could clearly see that there were more giant cells in the root knots of *Cshy5* and they occupied a larger area in the whole section at 7 (Figure S4a,b), 14 (Figure 3d,e), and 21 DAI (Figure S4c,d). The absolute counts of gall numbers and total nematodes in the root systems demonstrate that the loss of CsHY5 increases plant susceptibility to nematode infection (Figures S1 and S2).

### 3.2. The Nematodes That Invaded Cshy5 Mutants Developed to the Female Stage More Quickly

**Figure 4:**
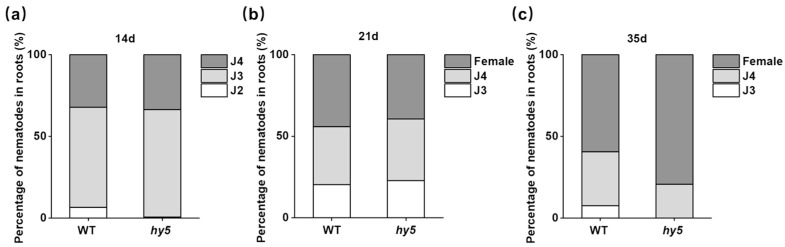
Developmental dynamics of nematodes in the root system. (**a**) Proportion of J2s, J3s, and J4s in the roots of *Cshy5* mutants and WT plants at 14 DAI. *p* = 0.366. (**b**) The proportion of J3s, J4s, and females in the roots of *Cshy5* mutants and WT plants at 21 DAI. *p* = 0.820. (**c**) The proportion of J3s, J4s, and females in the roots of *Cshy5* mutants and WT plants at 35 DAI. *p* = 0.017. (Percentage data were analyzed by a chi-squared test).

After invading the host root system, nematodes establish feeding sites within it and commence their development. The proportion of nematode population at various age stages reflects the developmental process of nematodes in the root system during their life cycle. Therefore, we calculated the proportion of the number of nematodes of different ages at 14 DAI, 21 DAI, and 35 DAI (Figure 4a–c). We found that in *Cshy5* mutants, J2s developed more rapidly to J3 and J4 at 14 DAI, and more J3s and J4s developed to the female stage at 35 DAI. The developmental dynamics indicated that, in *Cshy5* mutants, nematodes faced less hindrance in their development and could more easily complete their life cycle.

**Figure 5:**
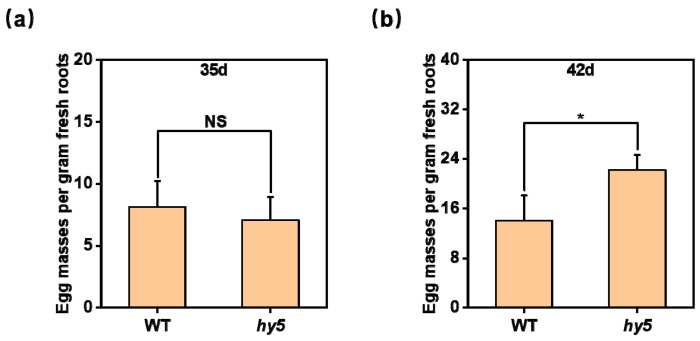
Egg masses of nematodes in the root system. (**a**) Number of eggs in the roots of WT and *Cshy5* mutants at 35 DAI. (**b**) Number of eggs in the roots of WT and *Cshy5* mutants at 42 DAI. The results are presented as the means ± SD; n = 6. Asterisks represent significant differences between *Cshy5* and WT (NS, no significance; *, *p* < 0.05; Student’s *t*-test).

To further investigate whether CsHY5 influences the capacity of mature female nematodes to produce egg masses, we enumerated the number of egg masses in the root system at 35 and 42 DAI. At 35 DAI, there was no difference in the number of egg masses in the root between WT and *Cshy5* mutants (Figure 5a). Therefore, we extended the count to 42 DAI to determine the number of egg masses in the root system. At 42 DAI, the number of egg masses in the *Cshy5* mutants’ root system was significantly higher than that in WT (Figure 5b). The absolute counts of egg masses in the root system demonstrate that the loss of CsHY5 leads to the formation of more egg masses in the roots (Figure S3).

### 3.3. Changes in Antioxidant Enzyme Activity and Hormones

**Figure 6:**
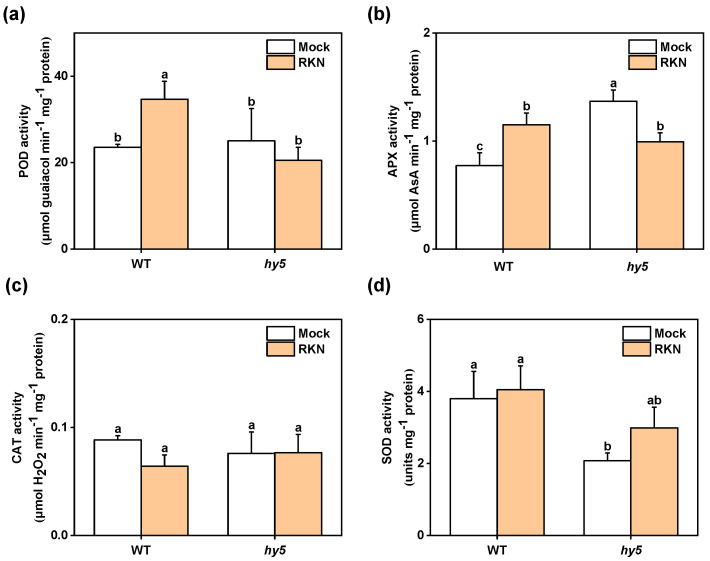
The effect of RKN on leaf enzyme activity in WT and *Cshy5* mutants after infection. (**a**–**d**) The activities of POD, APX, CAT, and SOD antioxidant enzymes in the leaves of infected and uninfected WT and *Cshy5* mutants 21 days after RKN infection. The results are presented as the mean ± SD; n = 3. Different letters indicate significant differences (*p* < 0.05) according to Tukey’s test.

Given the crucial roles of hormones and ROS in plant defense against RKNs, we further collected leaves from *Cshy*5 mutants and WT plants that were either infected or non-infected with RKNs at 21 DAI. We then measured the activities of four antioxidant enzymes: POD, APX, CAT, and SOD (Figure 6). By analysis, it was found that POD enzyme activity was induced in WT but not in *Cshy5* mutants (Figure 6a). The APX enzyme activity was similarly induced in WT, while it was significantly decreased in *Cshy5* mutants (Figure 6b). Interestingly, the CAT enzyme activity was still not induced in *Cshy5* mutants during RKN infection but showed a significant downward trend in WT (Figure 6c). However, SOD activity could not be induced in either *Cshy5* mutants or WT (Figure 6d). Surprisingly, the activities of these three enzymes (POD, CAT, and SOD) were not induced in *Cshy5* mutants during the RKN challenge. Moreover, the CAT enzyme activity in WT, as well as APX enzyme activity in *Cshy5* mutants, was not induced during nematode attack on plants, but showed a downward trend. Taken together, we can determine that antioxidant enzymes are involved in plant defense during nematode infection in cucumber and are not activated in mutants.

**Figure 7:**
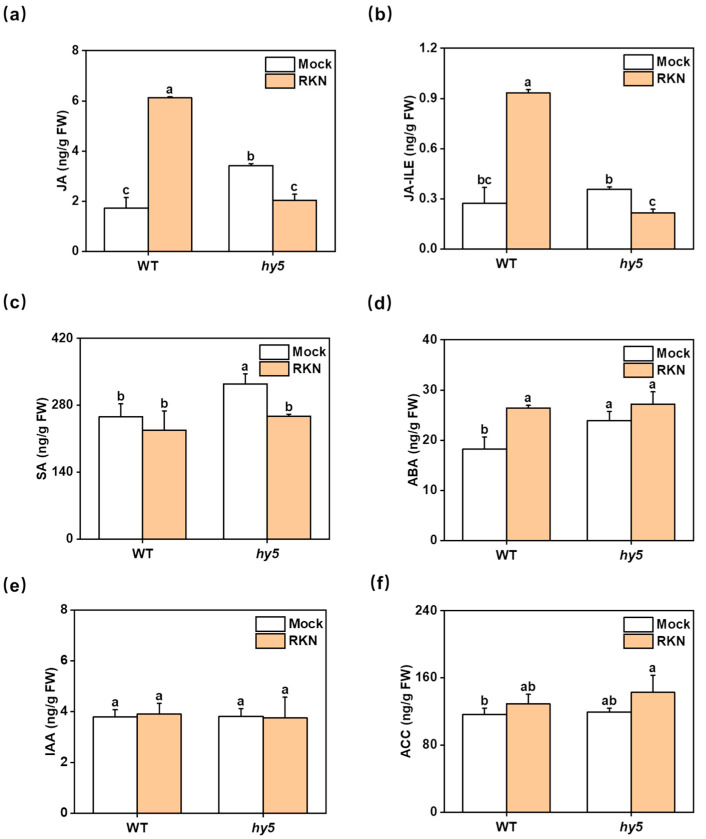
The effect of RKNs on leaf hormones in cucumber after infection. (**a**–**f**) The contents of JA, JA-Ile, SA, ABA, IAA, and ACC hormones associated with RKN defense in leaves of infected and non-infected WT and *Cshy5* mutants 7 days after RKN infection. The results are presented as the mean ± SD; n = 3. Different letters indicate significant differences (*p* < 0.05) according to Tukey’s test.

According to previous studies, various endogenous hormones in plants are implicated in the defense process during root-knot nematode infection of plant roots. Although root-knot nematodes initially infect the roots of plants, Wang et al. [29] found that plants’ RKN resistance is largely dependent on hormones in shoots. Therefore, we measured the contents of relevant hormones in the leaves. We chose to measure the contents of JA, SA, ABA, and IAA as well as ACC, the synthetic precursor of ethylene (Figure 7). We found that the increase in JA was more pronounced in WT, while it was significantly reduced in *Cshy5* (Figure 7a). The content of Ja-Ile was further determined, and the trend of Ja-Ile was found to be similar to that of JA (Figure 7b). A significant increase in ABA content was also observed, but no significant change was observed in *Cshy5* (Figure 7d). SA was decreased in both WT and *Cshy5* plants and significantly decreased in *Cshy5* plants (Figure 7c). The contents of IAA and ACC, the precursors of ethylene synthesis, did not change significantly (Figure 7e,f). Under RKN infection, the contents of hormones increased in WT and decreased or remained unchanged in *Cshy5*, which was consistent with the changes of antioxidant enzyme activities.

### 3.4. Transcriptome Analysis of WT and Cshy5 Infected with RKNs

The number of root knots, nematodes in roots, egg masses, developmental dynamics of nematodes in roots, hormone content, and antioxidant enzyme activity all indicated that deletion of the *CsHY5* gene enhanced cucumber sensitivity. To further investigate the mechanism by which CsHY5 deficiency leads to increased sensitivity of cucumber to root-knot nematodes (RKNs), we performed transcriptome sequencing on infected and non-infected WT and *Cshy5* mutant plants.

**Figure 8:**
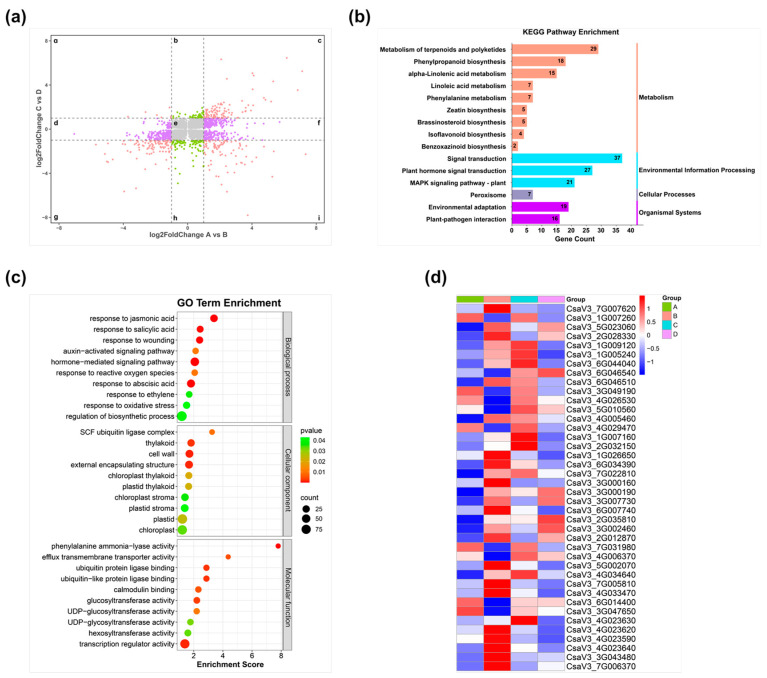
Analysis of transcriptome results. (**a**) Joint analysis diagram. log_2_FoldChange ≥ 1: up-regulation; log_2_FoldChange ≤ −1: down-regulation; |log_2_FoldChange| < 1: no change. (**b**) KEGG enrichment pathway map. (**c**) GO term enrichment map. (**d**) Heat map of associated gene expression. A: WT + Mock; B: WT + RKN; C: *Cshy5* + Mock; D: *Cshy5* + RKN.

To screen for differentially expressed genes exhibiting divergent expression trends between the two materials (WT, *Cshy5*) due to CsHY5 deficiency during RKN infection, we first compared transcriptome data before and after infection between the two materials. A threshold of *p* ≤ 0.05 was applied to filter genes with reliable results. The two sets of filtered genes were then compared to obtain their intersection. After identifying the intersection genes, we plotted them based on their log_2_foldchange values (Figure 8a). The plot enabled clear visualization of each gene’s expression trend in the two materials. We excluded genes showing identical expression trends in both WT and *Cshy5* groups: genes falling into regions c, e, g. Focus was placed on genes in six regions: a, b, d, f, h, i.

**Figure 9:**
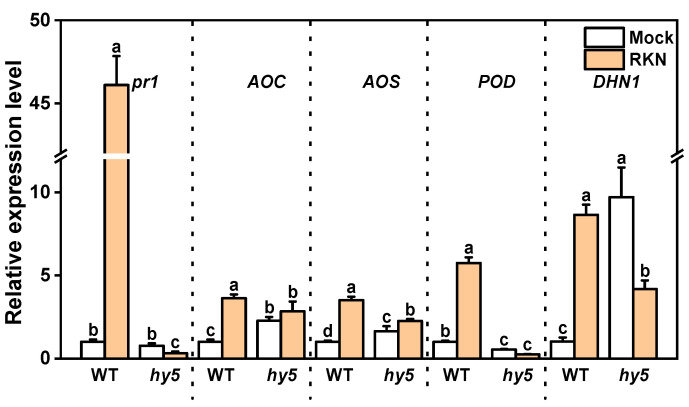
qRT-PCR validation figure. Relative expression values for each gene in different treatments are expressed as 2^−ΔΔCt^ values with WT + MOCK as 1. Error bars represent the SD of three biological replicas. Different letters indicate significant differences (*p* < 0.05) according to Tukey’s test.

In order to disclose the functional mechanism of these genes, GO and KEGG enrichment analyses were carried out on the genes in these six regions (Figure 8b,c). In the GO enrichment analysis, these genes were enriched in terms of hormone-mediated signaling pathway, response to hormone and phenylalanine ammonia lyase activity. The KEGG enrichment analysis indicated that these genes were enriched in pathways like the MAPK signaling pathway-plant, alpha-Linolenic acid metabolism, Linoleic acid metabolism, Phenylpropanoid biosynthesis, Peroxisome, Plant–pathogen interaction, and others. Based on the GO and KEGG enrichment results, the expression of some genes related to the response to wounding and the response to oxidative stress, and hormones were listed with heat maps (Figure 8d). To validate the reliability of the transcriptome results, we chose several key genes related to the plant’s resistance to RKNs and verified them through qRT-PCR. These included JA key synthase genes *CsaV3_5G023060* (AOC) and *CsaV3_2G028330* (AOS), the ABA-responsive gene *CsaV3_4G005460* (DHN1), the POD enzyme gene *CsaV3_4G023590*, and the pathogenesis-related protein gene *CsaV3_7G007620* (PR1). The results were consistent with the trends of the transcriptome data (Figure 8d), indicating the high reliability of the transcriptome results (Figure 9). Based on the transcriptome data, it can be determined that the loss of CsHY5 during RKN infection affects the transmission of infection signals and the response of the defense response.

## 4. Discussion

RKNs possess special biological characteristics and survival mode and have long been difficult to eradicate by the commonly used control methods. Traditional chemical control can merely exert a certain inhibitory effect in a short period, and RKNs tend to develop chemical resistance over time. Most physical prevention and control approaches are cumbersome to implement, costly, and difficult to apply widely. Although biological control methods are relatively environmentally friendly, they also have issues such as unstable control effects [57]. Under such a predicament, it is undoubtedly a feasible solution to cope with the harm of RKNs at present by enhancing the resistance of plants themselves and strengthening the ability of plants to resist the invasion of RKNs, thereby effectively reducing the adverse impacts of RKNs on plant growth, development, yield, and quality. As an important transcription factor in the process of light signal transduction, HY5 can integrate various pathways such as light signal, hormones, and so on, thus playing a role in the process of plant stress resistance [46]. Therefore, it is particularly important to study its role in cucumber defense against RKNs.

HY5 has been confirmed to play a critical role in plants’ responses to multiple stresses. It has been demonstrated that HY5 can promote the expression of *ABI5* by directly binding to its promoter region, disrupting the inhibitory effects of histone demethylase JMJ17 and the core transcriptional repressor WRKY40 of the ABA signaling pathway on *ABI5*. This mechanism enhances the adaptability of Arabidopsis to abiotic stresses such as drought and high salinity [58]. Additional studies show that drought conditions significantly reduce the expression of *ZmHY5*, thereby delaying maize flowering time as a coping strategy against drought stress [59]. In *Arabidopsis*, HY5 acts as an integration node for light and ABA signaling pathways, significantly enhancing the plant’s water reabsorption capacity under drought conditions by regulating root tropisms (gravitropism and hydrotropism) [60]. In our study, we confirmed that CsHY5 is involved in the interaction between cucumber and RKNs: the loss of CsHY5 led to more root knots and nematodes in the roots, enhancing cucumber sensitivity to RKNs. Moreover, CsHY5 deficiency also enabled RKNs to complete their life cycle more rapidly, reaching the female stage faster and producing more egg masses. Notably, a recent study in Arabidopsis demonstrated that HY5 directly binds to *AtSWEET11*, *AtSWEET12*, and *AtSWEET15* to negatively regulate nematode resistance, which contrasts with our finding of positive regulation in cucumber [61]. This discrepancy may arise from divergence in HY5 downstream signaling pathways across species: in cucumber, HY5 likely targets antioxidant enzyme- and hormone-related pathways more directly, whereas in *Arabidopsis*, HY5 regulatory networks may be coupled with root development or other defense pathways.

The crucial roles of hormones and ROS in plant defense against RKNs have been extensively validated. Several studies have shown that exogenous application of JA or its volatile derivative methyl jasmonate can enhance resistance to RKNs in multiple plant species, including tomato, rice, soybean, and *Arabidopsis* [62,63,64,65,66]. *Arabidopsis* mutants lacking JA biosynthesis genes such as *AtAOS* and *AtAOC* are more sensitive to RKNs, while mutants lacking the 13-lipoxygenase (*AtLOX3*) gene involved in JA biosynthesis exhibit reduced sensitivity to RKNs, indicating a complex role of JA in plant defense pathways against root-knot nematodes [63,67]. Deletion of NADPH oxidase genes (*RbohD* and *RbohF*) also renders plants more susceptible to RKN infection. In tomato, total antioxidant enzyme activity slightly increases in RKN-susceptible plants after infection, whereas it significantly increases in resistant plants [68,69]. In this study, hormone and antioxidant enzyme activity assays showed that JA, JA-Ile, ABA, POD, and APX were significantly induced in WT plants after RKN infection, with JA exhibiting the highest degree of change consistent with previous findings. By contrast, these parameters showed significant decreases or no changes in *Cshy5* mutants, suggesting that CsHY5 regulates hormone accumulation and antioxidant enzyme activation, thereby modulating plant sensitivity to RKNs. Transcriptome analysis also revealed that the deletion of CsHY5 led to the suppression of key JA biosynthesis enzyme genes *AOC*, *AOS*, and *POD* following nematode infection. Cluster analysis further indicated that these genes were closely associated with resistance responses after RKN invasion. This supports the conclusion that CsHY5 mediates cucumber resistance to RKNs by directly or indirectly regulating the antioxidant system and JA signaling pathway. Notably, the basal contents of JA and ABA and the activity of APX in Cshy5 plants were higher than those in WT plants in the absence of root-knot nematode infection. The genes related to JA and ABA also showed the same trend. This might be because CsHY5 has multiple regulatory modes for these pathways, and the specific regulatory modes require further investigation.

## 5. Conclusions

In summary, CsHY5 played a significant role in the resistance of cucumber to RKNs. The *CsHY5*-deficient material presented more galls and nematodes. Furthermore, *CsHY5* deficiency affected the activities of defense enzymes and hormones to varying extents. Thus, we propose that CsHY5 may regulate cucumber resistance to RKNs by affecting the response of cucumber to RKNs and the activation of defensive-related pathways.

## Data Availability

The original contributions presented in this study are included in the article and Supplementary Materials. Further inquiries can be directed to the corresponding authors.

## Supplementary Materials

The following supporting information can be downloaded at https://www.mdpi.com/article/10.3390/antiox14060679/s1.
