# Supplementary material for: ELONGATED HYPOCOTYL5 Regulates Resistance to Root-Knot Nematode by Modulating Antioxidant System and Jasmonic Acid in Cucumis sativus
Source: Antioxidants (Basel). 2025 Jun 3;14(6):679. doi: 10.3390/antiox14060679 (PMC12189740; doi:10.3390/antiox14060679)
Supplement: Supplementary file 1 [file antioxidants-14-00679-s001.zip › antioxidants-3544061-supplementary.pdf]

Table S1. Primers used for in this study

Primer name	Sequence (5'-3')
**Primers for CRISPR/Cas9 construct**	
CsHY5-AtU3d-F	GTCACTCGAGCGCATACGACTGA
CsHY5-AtU3d-R	AAACTCAGTCGTATGCGCTCGAG
CsHY5-AtU3b-F	GTCAGTAGAGTTCCGCAGATCTG
CsHY5-AtU3b-R	AAACCAGATCTGCGGAACTCTAC
CsHY5-AtU6-1-F	ATTGCCGGAGTTGCCGCAGATCTG
CsHY5-AtU6-1-R	AAACCAGATCTGCGGCAACTCCGG
CsHY5-AtU6-29-F	ATTGAAGGAGCAGAGGACGAAGTG
CsHY5-AtU6-29-R	AAACCACTTCGTCCTCTGCTCCTT
**Primers for RT-qPCR**	
Cspr1-F	GGACCTGTCAGTTGGGATGA
Cspr1-R	CCCATATGGCCCGTTAGAGT
CsAOC-F	TCAGAAGCGGCTCGGCATA
CsAOC-R	CGGTAACGGCTAAGTAGGTGTC
CsAOS-F	CCCGAAACTCAATCACCGTC
CsAOS-R	GAAGGAATCGTAGCGGAGGA
CsPOD-F	TGCTCTATCAGGTGCACACA
CsPOD-R	ATGTGCCTGACCCTGATTGA
CsDHN1-F	CGGACTGATGATGTGTGTTCGG
CsDHN1-R	CCGAGGAGCTAGAACTGGAG

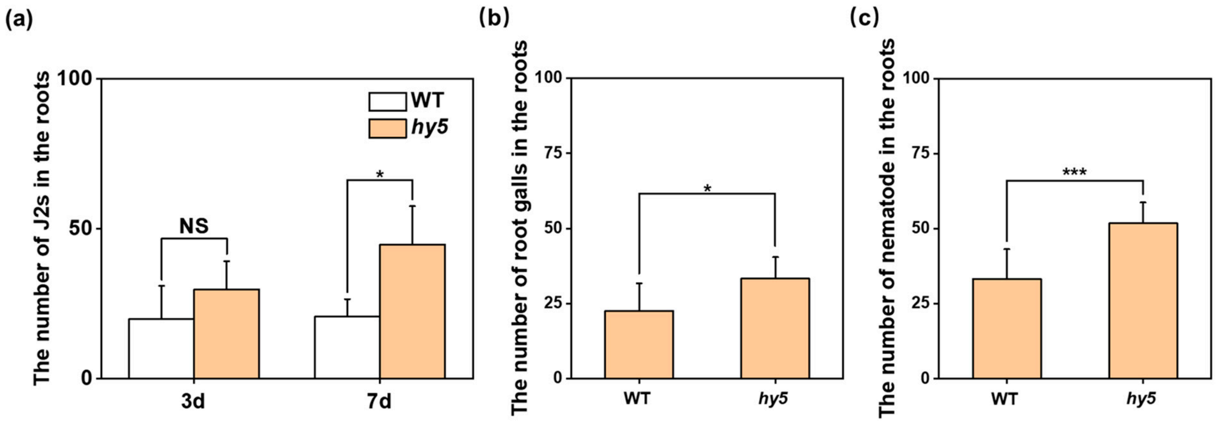

Figure S1. The loss of CsHY5 affects the susceptibility of plants to RKNs. (**a**) Number of J2s within the roots of WT and *Cshy5* mutants at 3 DAI and 7 DAI. (**b**) Number of root galls in WT and *Cshy5* mutants at 7 DAI. (**c**) Number of total nematodes in the roots of WT and *Cshy5* mutants at 7 DAI. The results are presented as the means ± SD; n = 6. Asterisks represent significant differences between WT and *Cshy5* mutants (NS, no significance; *, $p<0.05$; ***, $p<0.001$; Student's t-test).

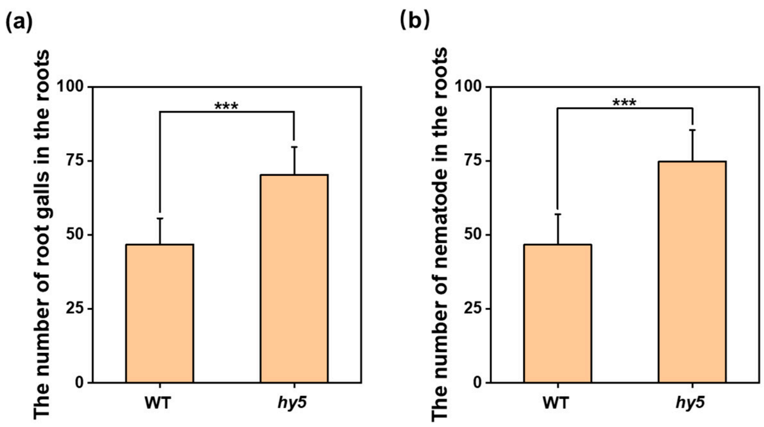

Figure S2. The loss of CsHY5 affects the susceptibility of plants to RKNs. (**a**) Number of root galls in WT and *Cshy5* mutants at 14 DAI. (**b**) Number of total nematodes in the roots of WT and *Cshy5* mutants at 14 DAI. The results are presented as the means ± SD; n = 6. Asterisks represent significant differences between WT and *Cshy5* mutants (***, $p < 0.001$; Student's t-test).

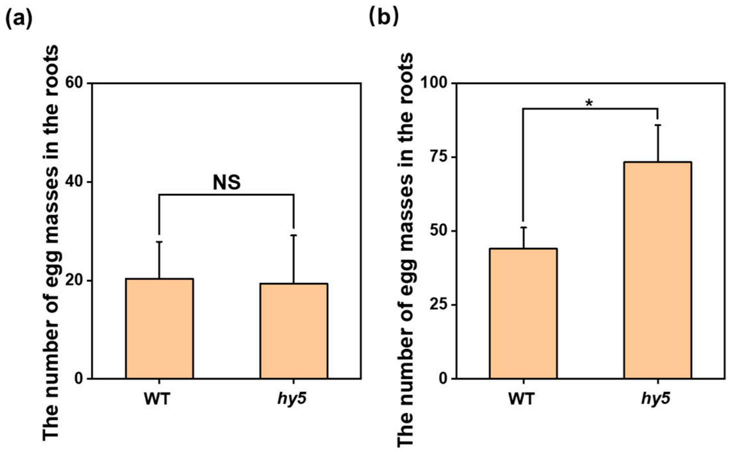

Figure S3. Egg masses of nematodes in the root system. (**a**) Number of eggs in the roots of WT and *Cshy5* mutants at 35 DAI. (**b**) Number of eggs in the roots of WT and *Cshy5* mutants at 42 DAI. The results are presented as the means ± SD; n = 6. Asterisks represent significant differences between WT and *Cshy5* mutants (NS, no significance; *, $p < 0.05$; Student's t-test).

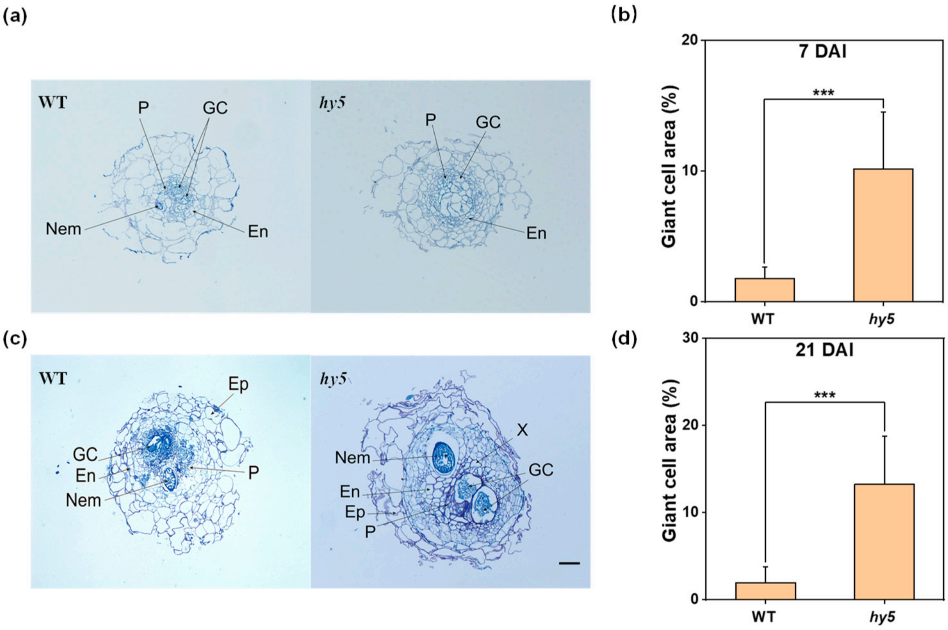

Figure S4. Gall sections and relative area of giant cells. (**a**, **c**) Anatomical structure of root knots in WT and *Cshy5* mutants root systems at 7 DAI and 21 DAI. (**b**, **d**) Area occupied by giant cells in WT and *Cshy5* mutants root knots at 7 DAI and 21 DAI. The results are presented as the means ± SD; n = 18. Asterisks represent significant differences between WT and *Cshy5* mutants (Nem, nematode; GC, giant cell; En, endodermis; P, phloem; Ep, epidermis; X, xylem) (The scale bar is 100 μm; ***, $p<0.001$; Student's t-test).
